# The Immune System Can Hear Noise

**DOI:** 10.3389/fimmu.2020.619189

**Published:** 2021-02-18

**Authors:** Andi Zhang, Tianyuan Zou, Dongye Guo, Quan Wang, Yilin Shen, Haixia Hu, Bin Ye, Mingliang Xiang

**Affiliations:** ^1^ Department of Otolaryngology & Head and Neck Surgery, Ruijin Hospital, Shanghai Jiao Tong University School of Medicine, Shanghai, China; ^2^ Ear Institute, Shanghai Jiao Tong University School of Medicine, Shanghai, China; ^3^ Shanghai Key Laboratory of Translational Medicine on Ear and Nose Diseases, Shanghai Jiao Tong University School of Medicine, Shanghai, China

**Keywords:** noise, immune function, immune diseases, noise-induced hearing loss, music

## Abstract

As a stressor widely existing in daily life, noise can cause great alterations to the immune system and result in many physical and mental disorders, including noise-induced deafness, sleep disorders, cardiovascular diseases, endocrine diseases and other problems. The immune system plays a major role in maintaining homeostasis by recognizing and removing harmful substances in the body. Many studies have shown that noise may play vital roles in the occurrence and development of some immune diseases. In humans, both innate immunity and specific immunity can be influenced by noise, and different exposure durations and intensities of noise may exert various effects on the immune system. Short-term or low-intensity noise can enhance immune function, while long-term or high-intensity noise suppresses it. Noise can lead to the occurrence of noise-induced hearing loss (NIHL) through the production of autoantibodies such as anti-Hsp70 and anti-Hsp60 and exert adverse effects related to other immune-related diseases such as some autoimmune diseases and non-Hodgkin lymphoma. The neuroendocrine system, mainly including the hypothalamic-pituitary-adrenal (HPA) axis and the sympathetic-adrenal-medullary (SAM) system, is involved in the mechanisms of immune-related diseases induced by noise and gut microbiota dysfunction. In addition, noise exposure during pregnancy may be harmful to the immune system of the fetus. On the other hand, some studies have shown that music can improve immune function and alleviate the adverse effects caused by noise.

## Introduction

Noise is an important environmental factor that affects human health. In general, any sound that causes irritability, stress, or disturbance on our normal life can be classified as noise. According to the WHO, by 2011, noise pollution had already become the most important environmental threat to public health after air pollution (1, 2).

Recently, an increasing number of studies on noise have been carried out, and the harmfulness of noise to the human body has gradually become recognized. With regard to hearing impairment, noise above 85 dB can cause permanent damage to the hair cells in the cochlea and eventually result in hearing loss, according to the National Institute on Deafness and Other Communication Disorders (NIDCD). In addition to affecting the auditory system, increasing evidence in recent years has confirmed that noise may cause various diseases, such as obesity, coronary atherosclerotic heart disease, hypertension, diabetes, sleep disorders, and mental illness (1, 3). Data from the WHO have shown that noise above 55 dB at night is hazardous to public health (2). Although the direct relationship between noise and the immune system is unclear, a few studies have recently shown that noise can cause some changes in the immune system (4), including proliferation of immune cells, secretion of cytokines, production of antibodies, and immunotoxicity, by regulating the HPA axis and other neuroendocrine regulatory axes (5, 6). Here, we review the progress regarding the impact of noise on the immune system from the following perspectives: the relationship between noise and the immune system, the correlations between noise and immune diseases, the mechanisms by which noise causes immune system disorders, the intergenerational influence of noise and the beneficial effects of music on the human body.

## Correlation Between Noise and the Immune System

The immune system is composed of multiple immune organs, immune cells and immunoactive substances. It can recognize and eliminate foreign pathogens, senescent cells and tumor cells in the body. Moreover, it can also maintain immune homeostasis through mediation of immunologic tolerance and immunomodulation. Many studies have shown that noise can affect innate and specific immune functions (3) and noise of different durations and intensities can elicit different immune responses (5, 7, 8). Sixteen studies (11 animal studies and five human studies, see **Table 1**) designed and analyzed a series of immunological alterations such as immunoglobulins, cytokines, and immune cells affected by noise exposure. Below, we summarize results of these studies according to the level of decibels.

**Table 1:** The immunological and hormonal adjustment after different decibels and durations of noise exposure.

Common environmental sounds	Decibels	Exposure measures	Experimental subjects	Main results-Immunological responses	Main results-Blood hormone levels
Ordinary conversation	50-70 dB	75-78 dB; 15 min (from 08:45 to 09:00) for 2 days	Beagle dogs 9.5±0.7 kg, 11 to 12 months old, N=10 (5 males and 5 females) (9)	-sIgA concentrations immediately after stress: dropped to less than half those of the controls (P=0.001); -sIgA concentrations at 30 min post-stressor: remained significantly lower (P=0.014). -D4sIgA concentrations at 60 min post-stressor: returned to near control levels.	
Heavy traffic	70-80 dB	peak sound levels: 85 dB; mean sound levels: 72 dB; exposure times: 1-4 days (A 2-h-long noise pattern of 69 aircraft noise events with a duration of 43 s)	C57B1/6j mice (10)	-Noise exposure led to a significant increase of myelomonocytic cells and natural killer cells within the vasculature on day 1 or 4; -No significant increase in infiltration was observed for neutrophils; -At least one significantly increased day for leucocytes in general as well as macrophages and monocytes; -No significant changes were observed for T cells.	-Plasma catecholamine levels (noradrenaline, dopamine significantly and adrenaline by trend) and angiotensin II levels increased; -Cortisol levels in urine showed no significant change (although a trend for increased values in response to noise exposure) and were increased in kidney after 4 days of noise exposure.
Noiy office	80-90 dB	85 dB; 15 min/h, 10h/d	Wistar male rats, 230-380 g, 3 months old (11)	Splenic NK cells activity showed a bimodal response to chronic noise exposure compared to control group. NK activity was increased after both 24 h and 7 days of exposure, whereas a decrease was observed after 3 weeks of noise exposure; -The phagocytic activity of both total granulocytes and the monocytes was significantly reduced after 1 day of noise exposure. After 7 or 21 days, however, the phagocytic activity in noise-exposed animals was not different from that in control group.	
		90 dB; group A:5 h (from 22:00 to 3:00) each day for 3 consecutive days; group B: 4 weeks (28 d) with the same exposure protocol	BALB/c male mice, 20-25 g, 6 weeks old, N=10 (7)	-The serum IgM in group A significantly increased (P<0.01) compared to control group; the serum IgG in group B significantly increased in group A (P<0.01) but significantly decreased in group B (P<0.01) compared to control group, although CD8+ cells were not significantly changed.	-Three days of exposure to noise stress resulted in a 2.5-fold increase of corticosteroid (5.58 μg/dl, p<0.01) and a 1.5-fold increase of adrenaline (1756.9 pg/ml, p<0.01); -Corticosteroid in group B was increased from 2.08 to 4.25 μg/dl(p<0.01) and adrenaline was increased from 792.8 to 954.9 pg/ml (p<0.05) as compared to control group.
		90-95 dB; 1-h period for 7 days	Female Wistar rats, N= 98 (12, 13)	-Noise exposure was associated with statistically significant increases in the prevalence of arthritis (p<0.05) and early in its course, in the severity (p<0.001) of arthritis; - There are few other existing data for evaluating the relationship.	
Noisy bar	90-100 dB	95-97 dB;1 h/day for 14 days	Inbred female BALB/c and C57B1/6J (C57) mice, 23-25 g, sixty days old (8)	-IgG antibody production (anti-SRBC IgG) after T-dependent antigen immunization decreased (P<0.05) in C57 mice; -Mitogen-induced proliferation significantly decreased (P<0.01) among lymphocytes from C57 mice exposed to noise.	-Serum corticosterone levels and splenic catecholamine content decreased in BALB/c mice but not in C57 mice.
World series domed stadium		100 dB; 4 h	Wistar strain male albino rats, 150-200g, N=15 (14)	-The antibody titre decreased (P<0.001) compared to control group; -The thymus weight and count increased (P<0.001) and the spleen weight and count decreased (P<0.001) compared to control group.	-The plasma corticosterone level was significantly increased (P<0.001) in the acute noise stress group.
		100 dB;1 min/h for 3 h for at least 3 nights	AKR and C57/BI6 male mice, 7 to 12 weeks old (15)	-Mitogen-induced proliferation of lymphocytes suppressed, while enhanced with longer exposure to sound stress.	-Plasma cortisol levels in similarly stressed C57/BI6 mice increased; -Daily exposure to the sound stress for more than 10 days produces an adaptation which brings cortisol to baseline levels.
Propeller plane	100-110 dB	100 dB;1 h for 20 days	Wistar albino male rats, 180-200 g (16)	-The noise stress significantly decreased the antibody titer; phagocytic index in noise stress induced animals was significantly decreased (0.007± 0.001); -The expression of NK cell cytotoxic protein (Granzyme B, Perforin) and TNF-α significantly decreased in the noise stress induced group animals; -The noise stress animal liver and spleen tissues has showed fragmented DNA due to DNA damage; -The expression of TNFα level reduced in noise stress group; -The noise stress induced group showed irregular liver architecture such as granulated cytoplasm and small uniform nuclei patterns, which refers to damage on cytoplasm; Histopathological observation of spleen showed enlargement of white pulp and irregular architecture of red pulp.	
Rock concert	110-120 dB	100 dB;4 h/day for 30 days	Male Wistar rats, 200-220 g, N=64 (17)	-Chronic noise exposure induced significant changes in the gut microbiota population at the phylum and genus levels; -Noise exposure induced increases in immunoglobulin (Ig)A, interleukin (IL)-1β and tumor necrosis factor (TNF)-α levels in the ileum that persisted for at least 3 to 7 days after noise cessation.	-Chronic noise exposure increase blood glucose and corticosterone (CORT) levels.
Jet aircraft	≥120 dB	120 dB or 150 dB, single 17-min LFN (low-frequency noise), 2-40 Hz; 120 dB or 150 dB, multiple 17-min LFN (five times a week during 13 weeks)	Wistar male rats, 170 ± 35 g, 12 weeks of age, N=96 (18)	-Both 120 and 150 dB LFN resulted in a 10-fold increase in the overall chromosomal aberrations range compared to the spontaneous chromosomal mutation rates in the bone marrow cells in rats; -LFN elevates the lmwDNA content in the blood plasma that may be associated with cell death; -A single LFN exposure significantly increases the lmwDNA content in the blood plasma, whereas multiple exposures continue to elevate lmwDNA levels; -LFN leads to a stable (at least 7 days) increase of lmwDNA in the blood plasma.	
		Road traffic noise (40-85 dB)	Cross-sectional study, 172 female subjects (5)	-As the road traffic noise increased by 1 dB, the percentage of NKT cells decreased by 0.038%, whereas the IL-12 level increased by 0.006 pg/mL; -The percentage of NK cells and INF-γ levels were not significantly associated with road traffic noise or sensitivity (P>0.05).	As the sensitivity level increased by 1 step, the cortisol level increased by 0.032 μg/dL.
		Road traffic noise (below 55 dB, between 55 and 65 dB, above 65 dB)	Case-control study, Danes between 30 and 84 years of age with a primary diagnosis of NHL between 1992 and 2010 (19)	-A 5-year time-weighted mean of road traffic noise above 65 dB was associated with an 18% higher risk for NHL (95% CI 1.01-1.37) compared to road traffic noise below 55dB. -No association between was found (odds) ratio: 0.98; 95% CI: 0.88-1.08) between 55 and 65 dB; -No association between road traffic noise and risk for T-cell lymphoma, whereas increased risks for B-cell lymphoma and unspecified lymphomas were observed at exposures above 65 dB.	
		80-85 dB (Never exposed, <5 years, 5-9 years, 10-14 years, and ≥ 15 years).	Cross-sectional study, a sample of 4192 participants (20)	-The main and fully adjusted models yielded OR=3.98 (95% CI: 1.74,9.11) and OR=2.84 (95% CI:1.23, 6.57) for participants exposed for ≥ 15 years compared to never exposed participants; -To conclude, long-term occupational noise exposure might be a modifiable risk factor for RA, but currently, the evidence base is very thin and tenuous. Future research in this is warranted.	
		75-115 dB	Cross-sectional study, 399 workers at the Dongfeng Motor Co in Shiyam, Hubei, China (21)	-The prevalence of positive anti-Hsp70 was significantly higher in the workers with slight and moderate high frequency (4-8 kHz) hearing loss than in normal workers (P<0.05); -The prevalence of positive anti-Hsp60 in workers with moderate low-frequency (0.5-2 kHz) hearing loss was significantly higher than in the normal (P<0.01)	
		Road traffic noise (residence ≤50 m from a highway and >150 m from a highway)	Nested case-control study 678,361 area residents (13)	-RA incidence was increased with proximity to traffic, with an odds ratio (OR) of 1.37 (95% CI: 1.11, 1.68) for residence ≤50 m from a highway compared with residence ≥50 m away. However, neither noise levels nor traffic-related air pollutants are responsible for this relationship.	

dB, decibel, used to measure sound intensity.

The quantities and activity of T-lymphocytes and immunoactive substances such as IgM are enhanced in mice after short-term noise exposure (7), which indicates that short-term noise exposure may cause temporary activation of immune function. This phenomenon is likely a compensatory mechanism, and the opposite effects of noise to the immune system increases as noise exposure is extended. Many researchers have found that the levels of immunoactive substances in the blood circulation, such as IgG and IgM (7), and of sIgA (9, 22) in the local mucosa are decreased after prolonged noise exposure. The quantities and activity of immune cells such as T-lymphocytes (8), phagocytes (23), NK cells (16), and NKT cells (5) are reduced. In addition, the weights of immune organs/tissues such as the thymus (24), bone marrow (18), and spleen (14) are significantly reduced, and even the genetic material of cells (18) in bone marrow exhibits obvious adverse changes (4). The above results indicate that long-term noise exposure can suppress immune function by downregulating various immune components. This is probably because noise not only induces the occurrence of certain immune diseases but also is correlated with several cancers. Recently, a few clinical studies have shown that the risks of breast cancer (25) and colorectal cancer (26) are increased in humans after exposure to long-term traffic noise. It is widely recognized that circadian disruption and stress caused by nocturnal noise are associated with breast cancer risk (27). However, there are only few studies related to noise and breast cancer, investigation of the correlation is still at an early phase (27). Therefore, new well-designed studies on this issue, including specific impact and potential mechanism, should be considered.

## Noise and Immune Diseases

### Noise-Induced Hearing Loss (NIHL)

Noise-induced hearing loss (NIHL), a major occupational hazard, is the most common sensorineural dysaudia after presbycusis. Once the intensity, frequency or duration exceeds a certain threshold, noise damages mainly hair cells in the cochlea, thereby inducing reversible or irreversible hearing loss. Recently, Wang et al. reported that the inflammatory response in auditory organ was involved in NIHL pathogenesis. Multiple inflammatory pathways were confirmed to have significant adjustment including the TNF signaling pathway, IL-17 signaling pathway, NF-κB signaling pathway, rheumatoid arthritis, and p53 signaling pathway (28). However, NIHL is not only induced through cochlear injury but also closely related to the production of some autoantibodies (29).

Heat shock proteins (Hsps) are a class of highly conserved proteins commonly expressed in organisms. In general, their expression levels in cells are low. Under conditions of high temperature and harmful stress, the synthesis rates of Hsps are increased rapidly to improve the organism’s ability to deal with stress. Hsps not only play roles as molecular chaperones but also play important roles in immune protection by stimulating humoral and cellular immunity (21).

Yang et al. reported that the levels of anti-Hsp antibodies in human plasma could be elevated by noise (21). After investigating the correlation of anti-Hsp60 or anti-Hsp70 with hearing loss in 399 workers exposed to noise between 75 and 115 dB, they found that the positivity rate of anti-Hsp70 was significantly higher in the plasma of workers with slight and moderate high-frequency (4-8 kHz) NIHL than in that of normal workers. Moreover, the positivity rate of anti-Hsp60 in workers with moderate low-frequency (0.5–2 kHz) NIHL was also significantly higher than that in normal workers (21). These results indicate that an elevated level of anti-Hsp70 in plasma is associated with a higher risk of high-frequency NIHL, whereas an elevated level of anti-Hsp60 in plasma is associated with a higher risk of low-frequency NIHL (21). These results suggest that anti-Hsp70 or anti-Hsp60 may play a role in the pathogenesis of NIHL and that different autoantibodies may cause hearing loss at different frequencies.

### Autoimmune Diseases

Autoimmune diseases are diseases caused by immune reactions against autoantigens in the human body, and the etiologies may be related to genetics, infections, hormones and other factors. Such diseases can lead to damage to multiple organs and systems mainly through the production of certain autoantibodies, and they include rheumatoid arthritis (RA), systemic lupus erythematosus (SLE), and multiple sclerosis (SS). In recent years, a few studies have demonstrated that noise may be a risk factor for several autoimmune diseases (12, 17, 20). In rats, 90–95 dB noise exposure for 7 days (1 h/day) is associated with increased severity of collagen-induced arthritis (12, 13), which suggests that noise is correlated with some autoimmune diseases.

Noise may be correlated with the onset of RA. Dzhambov et al. screened 4,192 participants exposed to occupational noise from 2011 to 2012 and analyzed the association of their exposure time with the prevalence of RA. They found that the prevalence of RA was higher among workers exposed to occupational noise for over 15 years compared to participants who were never exposed, and the fully adjusted model yielded an OR of 2.84 after exclusion of other risk factors such as air pollution (20). This result indicates that long-term occupational noise exposure might be a modifiable risk factor for RA. However, other scholars have argued that although an increased risk of RA might be related to the distance between an individual’s residence and the highway, noise is not a risk factor for RA (13) (OR≈1). We consider that other factors, such as noise sensitivity, heredity or sleep disorders, may be important factors leading to bias. Whether noise is related to the onset or progression of autoimmune diseases such as rheumatoid arthritis still needs further research.

Recently, some scholars have suggested that noise-induced sleep disorders increased the risk of autoimmune diseases, including RA, SLE and SS (3). The mechanism may be related to suppression of the activity of regulatory cells (Tregs) and production of several cytokines (30). Tregs are T cells that regulate autoimmunity, and sleep disorders can cause the occurrence of autoimmune diseases by decreasing the activity of Tregs. In addition, sleep disorders also increase the levels of cytokines such as IL-1, IL-1β, IL-6, IL-17, and TNF-α. Increases in IL-17 are associated with a variety of immune diseases, including RA, SLE and inflammatory bowel disease (IBD), through promotion of the production of certain autoantibodies (30).

In addition, noise may also indirectly cause autoimmune diseases by increasing smoking behavior. Some researchers have found that industrial noise exposure can result in increases in smoking behavior and the number of cigarettes smoked among workers (31, 32). Cigarette smoke contains hundreds of potentially toxic components, including tars, nicotine, carbon monoxide and polycyclic aromatic hydrocarbons. These substances contain high concentrations of free radicals, which can induce changes in genetic material and activation of related gene expression, thereby promoting the expression of various autoantibodies, such as rheumatoid factor, anti-cyclic citrullinated peptide antibody, and anti-dsDNA antibody (33). In addition, they can increase the expression of Fas/CD95 on B and CD4^+^ T lymphocyte cell surfaces and the sensitivity of those lymphocytes to apoptosis signals, further increasing the production of apoptotic material and the burden of material to be cleared. The events mentioned above eventually promote the development of various autoimmune diseases (33, 34). To date, five autoimmune diseases have been proposed to be related to smoking: RA, SLE, Graves’ disease, SS and primary biliary cirrhosis (33).

## Mechanisms by Which Noise Causes Immune System Disorders

Since noise may have positive or negative effects on multiple components of the immune system, how does noise exert its effects? To date, few studies on the relationship between noise and the immune system have been reported. Because the immune system is closely correlated with the endocrine system, circulatory system, respiratory system and other systems, it is possible that noise may affect the immune system *via* an indirect route (3, 8).

### HPA Axis and SAM System

**Figure 1:**
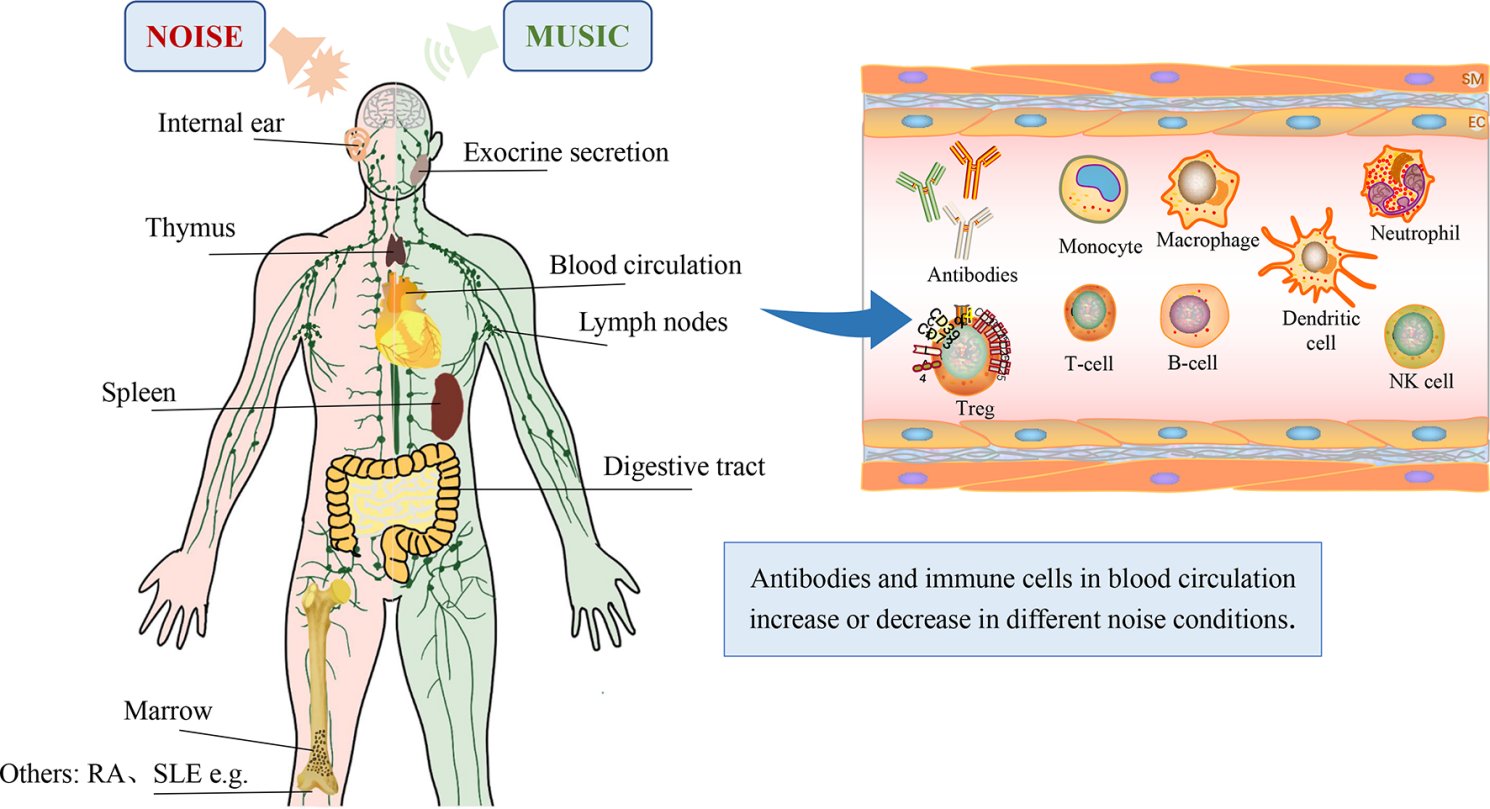
Possible mechanisms of the immunological effects of noise. We speculate that the influence of noise on the immune system may be correlated with neuroendocrine regulation such as the hypothalamic-pituitary-adrenal (HPA) axis and sympathetic-adrenal-medullary (SAM) system (3, 8), as well as oxidative stress (10) and sleep disorder (3, 27) which partly reveal the direct connections between noise and immunity. Changes in several inflammatory factors and immune cells (7, 17) have also been found.

Many studies have already shown that the neuroendocrine pathway is closely related to the mechanism by which noise affects the immune system (3, 35). Some neurotransmitters and endocrine hormones, such as ACTH, prolactin (PRL), somatropin (GH), thyroid-stimulating hormone (TSH), opioid peptides and dopamine, have been confirmed to be correlated with the regulation of immune function (6). As a stressor, noise can induce oxidative stress in the body and regulate the nervous system, circulatory system and endocrine system. It is known that some antioxidants, such as lipoic acid (36, 37) and *Scoparia dulcis* (38) can reduce changes in humoral and cellular immunity caused by noise. Therefore, we speculate that the influence of noise on the immune system may be correlated with neuroendocrine regulation and oxidative stress, although its specific mechanism remains to be explored. It is widely recognized that noise exposure can indirectly regulate the immune system by activating the HPA axis and SAM system (**Figure 1**) (8). A cross-sectional study revealed that noise exposure would affect cortisol levels in humans which increased by 0.032 µg/dl as the noise sensitivity level increased by one step (5). In addition, there were similar phenomena such as increased levels of plasma glucocorticoids and other endocrine hormones (noradrenalin, adrenalin, and angiotensin II) in mice exposed by noise as well as regulation of immunological substances (5, 7, 10). Therefore, neuroendocrine mechanisms such as the HPA axis may be involved in the noise-triggered adjustment of the immune system (3).

Recently, some scholars have proposed possible mechanisms of immune system activation induced by short-term noise. Short-term noise exposure increases the levels of norepinephrine, epinephrine, angiotensin II, and cortisol in the peripheral blood (5), and the accumulation of these hormones induces activation of endothelial NADPH oxidase, which can cause oxidative stress in the vascular system (10). The formation of endothelial superoxide may lead to uncoupling of nitric oxide synthase (eNOS) and reduce the production of NO by promoting oxidation of the eNOS essential cofactor BH4 to the BH3 radical and S-glutathionylation. Meanwhile, the levels of IL-6 and ET-1 in the vascular endothelium are also increased (10). These changes may promote the infiltration and aggregation of monocytes, NK cells and neutrophils in the vessels (5, 10). Eventually, the number and activity of various immune cells in the vessels increase, resulting in increased immune activity in a short time. In addition, researchers have found that the level of the NADPH oxidase subunit NOX-2 in the vascular endothelium is increased and have speculated that this increase may be secondary to inflammatory cell infiltration (10).

Some scholars believe that long-term noise may regulate the function of the immune system by chronically activating the HPA axis and the SAM system (6). Various immune cells, such as macrophages and T-lymphocytes, express glucocorticoid receptors (GRs). Long-term noise may result in chronic activation of the HPA axis, which can upregulate the levels of glucocorticoids (GCs). Binding of GRs with GCs in the cytoplasm of immune cells can prevent NF-κB from shifting to the nucleus, and GCs can then inhibit the expression of cytokine genes by activating NF-κB inhibitors, binding to NF-κB directly, or competing against GRs or NF-kB for binding to proteins such as CBP (cAMP response element binding protein) and SRC-1 (steroid receptor coactivator-1) (6). Eventually, the secretion of cytokines in lymphocytes is reduced, and immune function is suppressed. Moreover, chronic activation of the SAM system can lead to increases in catecholamine levels in the body. After binding to adrenaline receptors on the surfaces of immune cells such as lymphocytes and macrophages, catecholamine can induce the transcription of many cytokines by activating the cAMP-PKA signaling pathway in those cells (6), which eventually results in immune dysfunction. In addition to glucocorticoids and catecholamines, the neuropeptide substance-P can also suppress immune inflammation by inhibiting the secretion of IL-16 and other cytokines in eosinophils (6). These observations indicate that the neuroendocrine pathway may be associated with the effect of noise on the immune system.

### Gut Microbiota

There are a variety of bacteria in the human intestine that may affect digestive function, resist infection and change body weight, and they are even closely related to the risks of certain autoimmune diseases. Normally, the predominant microflora and the subdominant microflora maintain a steady state, thereby maintaining normal gut function. Many factors, such as environmental stressors, infection and genetic factors, can cause intestinal flora imbalance, which may lead to many diseases, including inflammation, obesity, autoimmune diseases, and even cancers (39).

Studies in rats have shown that chronic noise can change the intestinal inflammatory response and then lead to intestinal flora imbalance, such as conditions of increased Proteobacteria and Roseburia abundance or decreased Actinobacteria abundance (17). Proteobacteria include Enterobacteriaceae spp., Salmonella spp., *Vibrio cholerae*, *Helicobacter pylori* and other opportunistic pathogens. Upregulation of Proteobacteria may lead to increases in bacterial toxins in the intestine and ultimately cause intestinal inflammation, while Roseburia is associated with weight loss and impaired glucose tolerance (17). In addition, immunoglobulin (Ig)A, interleukin (IL)-1β, and tumor necrosis factor (TNF)-α levels are significantly increased in the ilea of noise-exposed rats, and the levels remain elevated for at least 3–7 days after cessation of noise exposure (17). Among these proteins, IgA not only participates in the intestinal innate immune response but also regulates the composition and function of the gut microbiota. An increase in IgA indicates a change in intestinal immunologic surveillance function and increases in inflammatory molecules, which may induce inflammatory bowel disease (IBD). Additionally, it has been confirmed that TNF-α can cause increases in triglyceride (TG) levels and very-low-density lipoprotein (VLDL) levels in rats and humans and inhibit the function of glucose transport stimulated by insulin, which eventually leads to insulin resistance (17). Previous studies have demonstrated that the intestinal microbiota changes significantly in patients with type II diabetes, exhibiting changes such as increased opportunistic pathogen abundance (17, 40). Therefore, these results suggest that chronic noise exposure may trigger the occurrence of metabolic diseases such as diabetes by inducing changes in the intestinal microbiota, which may be another mechanism in addition to the mechanism involving increases in glucocorticoid levels.

## Intergenerational Impact of Noise

The function of the human immune system may be changed by noise exposure; in addition, the harmfulness of noise may be hereditary and noise may have intergenerational effects on humoral immunity (35).

The serum IgG levels and thymus weights were lower in pregnant female mice randomly exposed to fire alarm noise of 85–90 dB for 1 h/day (30 sounds in total) every day during the 15–21 days of pregnancy than in controls (41). Moreover, after injection of herpes simplex virus-1 (HSV-1) antigen into the offspring of mice, the levels of specific antibodies in the serum were significantly lower than those in controls, and the Arthus reaction (AR) of the skin to old tuberculin was also significantly reduced after HSV-1 antigen injection (41). In addition, the splenic lymphocyte proliferation response of the mice was significantly enhanced by stimulation with pokeweed mitogen (PWM) and phytohemagglutinin (PHA), while it was inhibited by stimulation with concanavalin A (Con A) (41). These results indicate that exposure of mice to noise during pregnancy may affect the humoral and cellular immune functions of their offspring, which may be related to the development of the immune system or the hypothalamic-pituitary axis of the offspring mice. The specific mechanism needs to be confirmed by exhaustive research in the future.

## Music and Immune System

**Figure 2:**
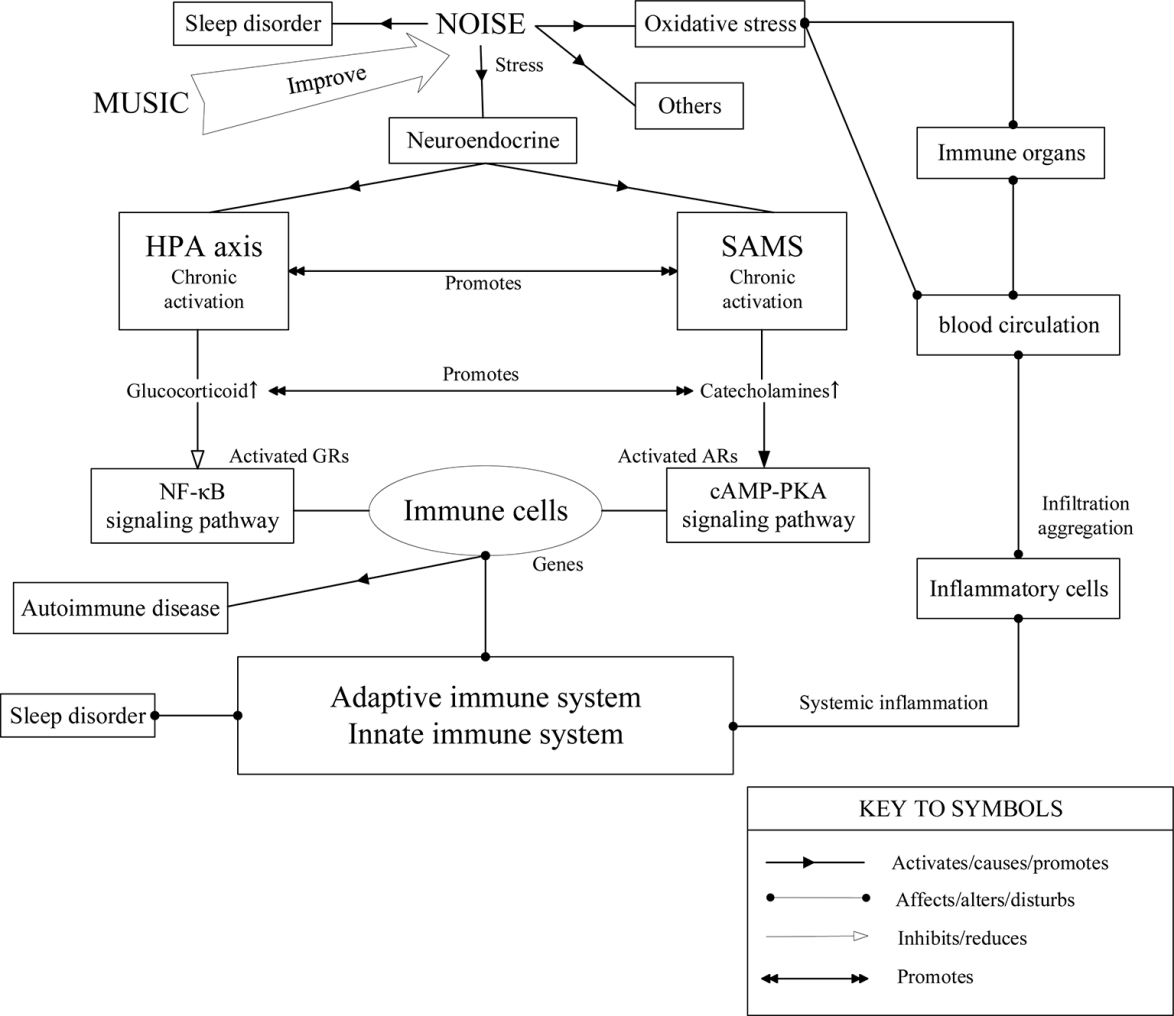
Changes in the immune system after exposure to noise or music. Most components, including the internal ear (21), exocrine secretions (9), thymus (24), lymph nodes (14), spleen (14), digestive tract (17), marrow (18), immunoglobulins (7, 8, 16) and immune cells (5, 7, 10, 11), are shown.

Every coin has two sides, and the good side of the sound coin is music (**Figure 2**). Music can activate the cerebral cortical area and produce a sense of pleasure, so music therapy can reduce and even eliminate psychological barriers through its unique physiological and psychological effects, thereby treating conditions such as cardiovascular diseases, dementia, epilepsy, and cancer pain and aiding in stroke rehabilitation (42, 43). Additionally, music can regulate the function of the immune system by reducing the hyperactivity of the HPA axis induced by stressors and eventually attenuate the suppression or enhancement of the immune system by noise (42).

Studies have shown that music can regulate immune function in healthy humans by enhancing the activity of NK cells, increasing the number of T-lymphocytes and promoting the production of IFN-γ and IL-6 (44). Furthermore, music can have an obvious reversing effect on the immunosuppressive effects caused by noise, including decreases in thymus and spleen weight and the number or activity of lymphocytes and NK cells. Music can also slightly improve these immune parameters in unstressed mice (42). Music can adjust immune function and alleviate the adverse effects of stressors on the immune system, which may be related to decreases in the levels of cortisol in the blood (44, 45). In particular, some evidence has indicated that music may even reduce distant metastasis of tumor cells in mice and postpone cancer progression (42).

In addition, music therapy may promote immune tolerance after organ transplantation, resulting in prolonged allograft survival. The numbers of CD4^+^CD25^+^Foxp3^+^ regulatory cells in the spleens of CBA mice were increased when the mice were exposed to classical music after allogeneic heart transplantation. At the same time, the levels of proinflammatory cytokines such as IL-2 and IFN-γ were suppressed, while those of anti-inflammatory cytokines such as IL-4 and IL-10 were increased, thereby promoting prolonged survival of the fully allogeneic cardiac allografts in CBA mice (46, 47). The results of these studies suggest that music therapy may have great significance for improving quality of life in clinical organ transplant patients.

## Prevention and Treatment

For the aforementioned changes in the immune system induced by noise, the prevention and treatment of noise are particularly emphasized. Maeda et al. reported that 31.0% (26/84) of genes in inflammatory and immune pathways were up- or downregulated in the cochleae with NIHL at 12 h post-noise exposure, including Ccl12, Ccl2, Ccl4, Ccl7, Cxcl1, Cxcl10, and Ptgs2 (upregulated genes), and Ccr7, Cxcr2, Kng1, Ltb, and Tnfsf14 (downregulated genes) (48). Moreover, systemic dexamethasone can improve the above changes partially, which may provide a basis for medicine treatment of NIHL (48, 49). Considering the extra-cochlear damage of immune substances caused by noise, antioxidants are known to be one of the effective therapeutic drugs currently. Srikumar et al. showed the supplementation with Triphala restores the changes in the antioxidant and cell-mediated immune response in rats exposed to noise, which may be due to its antioxidant properties (37). In addition, another study indicated that S. dulcis (SD) can prevent and even normalize the changes in cell-mediated and humoral immunity induced by noise stress in the Wistar rats, including the increase in corticosterone, soluble immune complex and IL-4 levels and the decrease in cytokines such us IL-2, TNF-α, IFN-γ (38). The result indicated the role of antioxidant, anti-stressor and immune stimulant activity of SD. Furthermore, other antioxidants also show similar therapeutic effects against noise such as vitamins A or E (tocopherols) and α lipoic acid (36).

However, the prevention strategies of noise are more significant and effective compared with the treatments after noise exposure. People must have a basic understanding of what risks caused by noise are involved above all, and wearing hearing protection (earplugs or earmuffs) and avoiding sources of hazardous noise such as the limitation of access to excessively noisy situations or purchasing volume-limiting headphones would be effective strategies (50). Additionally, soundproof wall is one of the measures for residents living around highways. For workers in noisy workplaces, the preventive measures against noise include periodic noise exposure monitoring in workplaces, engineering and administrative controls, personal hearing protection and routine audiometric examinations or health education before permanent damage to the inner ear (51).

## Conclusions

Noise can suppress or enhance immune function by changing the levels of innate immunity and specific immunity, and the changes in the immune system vary with the exposure duration and intensity of noise and with individual sensitivity. Overall, we can speculate that short-term or low-intensity noise may improve innate and specific immunity, while long-term or high-intensity noise suppresses the corresponding immune function. Noise-induced hearing loss (NIHL) is associated with the production of certain autoantibodies, and long-term occupational noise exposure may be a risk factor for rheumatoid arthritis. However, the direct correlations between noise and immune diseases remain to be confirmed due to the fact that few relevant studies have been performed.

In addition, the currently known mechanisms by which noise affects the immune system are related mainly to neuroendocrine pathways, such as the HPA axis and SAM system, which directly affect immune function. However, the molecular pathways underlying the mechanisms of the effects of noise on the immune system are still poorly understood. Whether and how noise can directly affect immune function is still worth investigating.

## Review Methodology

The literature searches were conducted in PubMed, Google Scholar and Web of science with the terms, “noise and immune”, “noise and immunity”, “noise and inflammation”, “noise and innate immunity”, “noise and adaptive immunity”, “noise and NK cell”, “noise and T cell”, “noise and B cell”, “noise and sIgA”, “noise and cytokines”, “noise and immunoglobulins”, “noise and IL”, “noise and autoimmunity”, “noise and cellular immunity”, “noise and humoral immunity”, “noise and thymus”, “noise and marrow”, “noise and spleen”, “noise and lymph nodes”, “noise and rheumatism”, “noise and diseases”, studies associated with music and protection were also collected and analyzed. Duplicated studies were excluded by examining the author list, abstract and results. The final review included 57 related literature.

## Author Contributions

MX: substantial contributions to the design of the work. AZ, BY, TZ, DG, QW, YS, and HH participated in the conception and design of the study, collected the literature, prepared the tables, and wrote the manuscript. The authors agree to be accountable for all aspects of the work in ensuring that questions related to the accuracy or integrity of any part of the work are appropriately investigated and resolved. MX: approval of the final version of this article. All authors contributed to the article and approved the submitted version.

## Funding

This work was supported by the National Natural Science Foundation of China (Grant No. 91949119 and 81670926) and Shanghai Sailing Program (Grant No.20YF1426400 and 19YF1430300).

## Conflict of Interest

The authors declare that the research was conducted in the absence of any commercial or financial relationships that could be construed as a potential conflict of interest.
